# Corrosion and Oxidation Behavior of a Fe-Al-Mn-C Duplex Alloy

**DOI:** 10.3390/ma12162572

**Published:** 2019-08-12

**Authors:** Silvia Barella, Andrea Francesco Ciuffini, Andrea Gruttadauria, Carlo Mapelli, Davide Mombelli, Eugenio Longaretti

**Affiliations:** 1 Dipartimento di Meccanica, Politecnico di Milano, via La Masa 1, Milano 20156, Italy; 2 F.G.S., Via Garzoneri 11, Treviglio (BG) 24047, Italy

**Keywords:** corrosion, Fe-Mn-Al-Ni steel, light steel, potentiodynamic curves

## Abstract

The low-density steels represent a topic of great interest within the scientific world because of the great demand from the steel market of increasingly lighter materials, also featured by an optimal mix of the mechanical properties. In this work, the corrosion and hot oxidation resistance of a Fe-15%Mn-9.5%Al-6.5%Ni-1%Cr-0.43%C were analyzed and related to the microstructural features. The material behavior was analyzed both in the as-cast and in the heat-treated state. For the corrosion test, the experimental plan was fulfilled using four different concentrations of HCl and four temperatures. In the case of hot oxidation resistance, the exposure time and the temperature effects were evaluated. The corrosion resistance in HCl was comparable to the stainless steel, and the iso-corrosion curves showed excellent resistance of the 1300 °C solution-treated material, especially at low temperatures, but it is also good at high temperatures due to the hot oxidation.

## 1. Introduction

New steel grades are continuously developed to optimize the properties, such as low-density, good mechanical properties, corrosion resistance, and high-temperature oxidation resistance. In particular, since the 1980s, the metallurgical system featured by Fe-Al-Mn-C has been investigated, and a great potential for substituting the Fe-Ni-Cr-C class has been discovered [1,2,3,4,5,6]. Nevertheless, up to now, the specific tensile strength (the ratio between the strength and the density) is lower than the one pointed by the Aluminum alloys or Titanium alloys [7,8,9]. Different studies have already demonstrated that the addition of Nickel in the Fe-Al-Mn-C class had beneficial effects increasing the specific yield strength [9]. Further, also Chromium resulted as a promoter of tensile strength and elongation to fracture in previous studies [7,10].

In this work, the interesting potential of the combined addition of Nickel and Chromium in low weight percentage to the Fe-Al-Mn-C class was analyzed. In particular, this study focused on wide assessment of the resistance to chemical degradation phenomena of the Fe-15%Mn-9.5%Al-6.5%Ni-1%Cr-0.43%C alloy (referred as MANC steel in the following sections of the paper, where M = Mn; A = Al, N = Ni, and C = Cr), that is a new steel grade. This work aimed at comparing this new steel grade to its natural rivals, i.e., commercial stainless steels, which show significant resistance to the chemical degradation phenomena that is one of their prominent features.

Among the chemical degradation phenomena, corrosion plays a predominant role. Corrosion is a predominantly surface damage phenomenon of metals, which leads to a loss in weight, a detachment of the material, and the consequent malfunction of components. The study of the corrosion resistance is a necessary step to compare directly the performances to the ones pointed out by the stainless steel grades, which are materials selected for many applications mainly to prevent or limit these degrading phenomena.

For metallic materials, the corrosion process is electrochemical and, therefore, it consists of a chemical reaction involving the transfer of the electrons when an electrolyte is present, and the reduction-oxidation reactions take place [11].

To derive the useful values of the corrosion resistance of this low-density steel grade, the determination of the iso-corrosion curves is needed because they allow a direct comparison to other steels grades, in particular, stainless steels. The iso-corrosion curve represents an effective tool for the evaluation of the general corrosion resistance of a metal or an alloy, taking into account the overall corrosive phenomena [12].

Corrosion in stainless steels is minimal but highly dependent on a few factors: the type of etchant and its concentration, the temperature, the impurities present in the medium, and their concentration. Generally, the higher the concentration of the etchant, the higher is the corrosion rate. One exception is the sulfuric acid, which for over 90% concentration reduces its corrosive power. Another key factor is the temperature: also, in this case, it is possible to conclude that generally the higher the temperature, the more severe will be the corrosion, irrespective of the type of the etchant solution. All types of corrosion are affected by temperature [13,14]. The impurities play an unfavorable role in passive metals, as might be deposited on the surface of the material, and they prevent proper development of the passive layer, and the consequence of such a situation could be an increase in the corrosion rate [13].

It is also possible to highlight the few elements that have a great impact on an increase in the corrosion resistance: Chromium, Nickel, Molybdenum, and Copper.

Finally, the austenitic phase is the most corrosion-resistant, essentially due to the higher Nickel concentration. Moreover, a low volume fraction of the non-metallic inclusions implies an improvement of the corrosion resistance: impurities are sites that may inhibit the formation of the passive film, and they may be preferentially corroded. Thus, the micro-purity of the material and its chemical and microstructural homogeneities play a key role to determine the corrosion resistance.

The achievement of good conditions for all these aspects represents a challenge for the developments of the new steel grades as the analyzed Fe-15%Mn-9.5%Al-6.5%Ni-1%Cr-0.43%C alloy. The response of the low-density MANC alloy against the corrosion phenomena was investigated in this work. 

On the contrary, the chemical corrosion, also defined as hot oxidation, is a degradation phenomenon of the materials that occur in gaseous environments at high temperature. It is also defined as dry corrosion and occurs in the absence of aqueous solutions. However, as for the wet corrosion, the process of formation of the oxide layer can be expressed by a reduction half-reaction coupled to an oxidation reaction [11]. The oxidation reaction leads to the formation of the metal ions,

$$M\to M^{2+}+\;2e^{-}$$

and it occurs at the metal-oxide interface. On the other hand, the reduction half-reaction produces oxygen ions,

$$\frac{1}{2}O_{2}+2e^{-}\to O^{2-}$$

and it occurs at the oxide-gas interface.

The rate of formation of the oxide layer is ruled by several factors, such as the nature of the atmosphere (gases, pressure), the nature of the metal (crystalline structure, chemical composition, melting temperature), and the oxide nature (state of aggregation, chemical composition, adherence). The relationship between the relative volumes of the oxide and the metal covers crucial importance. In general, the elements favorable in hot oxidation control are Chromium, Nickel, and Silicon [13].

The increase of the Chromium concentration increases the resistance to hot oxidation for the stainless steels. The austenitic stainless steels are the most exploited for the applications requiring a significant resistance to the hot oxidation [15].

The great expectations about the application of the low-density steels refer also to their possible exploitation as a reliable substitute of the stainless steels. So, these steel grades should be extremely resistant to corrosion and hot oxidation. Moreover, the low-density steels should rival with the austenitic stainless steels, which show excellent resistance at high temperatures [16]. The resistance to hot oxidation in air and at atmospheric pressure of the austenitic stainless steels for 24 h exposure time and a temperature range between 600 °C and 900 °C reaches a maximum value of 0.6 $\mathrm{mg}/{\mathrm{cm}}^{2}$ [17].

However, for a direct comparison with the hot oxidation rate, the limit value of the stainless steels has to be decreased since it is a function of the alloy density:

Hot−Oxidation Rate Limit=Hot−Oxidation Rate of Stainless Steels× Low−density alloy DensityStainless Steels Density=0.6× 7.367.9≅0.56mgcm2

Thus, to obtain competitive values of the hot oxidation rates if compared to the commercial stainless steels, MANC alloy would have a maximum hot oxidation rate limit of 0.56 $\mathrm{mg}/{\mathrm{cm}}^{2}$.

## 2. Materials and Methods

In this research, a Fe-15%Mn-9.5%Al-6.5%Ni-1%Cr-0.43%C alloy was melted in 10 kg electric arc furnace (FGS, Treviglio, Italy), using the same raw materials of the steel production cycle. The chemical composition of the alloy was checked through Optical Emission Spettroscopy measurements. Selected samples of the as-cast alloy underwent solution thermal treatment in an Argon-protected atmosphere for 1 h/inch at 1050 °C, 1200 °C, and 1300 °C and for 24 h/inch at 600 °C, 750 °C, and 900 °C. The cooling path was performed through a water quenching. For all the samples the assessment of the microstructures was achieved through specimen preparation, (referring to ASTM E3-11), chemical etching by the Beraha’s tint etching or by Nital 5% (ASTM E407-07), optical microscopy (Nikon, Tokyo, Japan) observations, and Scanning electron microscopy/energy dispersive spectroscopy SEM/EDS and SEM/EBSD (electron back-scattered diffraction) analysis with a Zeiss Evo 50 (Oberkochen, Germany) Any other data regarding the optical microscopy were obtained through the same method, and any data extrapolated from the images were processed through the NIS-Element software (Nikon, Tokyo, Japan). Further, any other data about the chemical composition were obtained through SEM/EDS analysis, expressed in wt.%.

The corrosion resistance in the chloride-rich environments and the hot oxidation resistance of this MANC alloy were measured and evaluated.

The corrosion resistance in the chloride-rich environments was established through the identification of a corrosion rate. The corrosion rate was expressed in millimeters per year. The same conversion table used by Oguike et al. [18] was used because it defines the conversion factor to derive the iso-corrosion curves from the quantities directly measurable. In particular, the needed data were easily acquirable as the change in weight, the time, and the surface exposed to the corrosive environment. Then, the conversion proposed by Oguike et al. [18] was used, and this provided the corrosion rate in mm/y, normalized on the density of the material. The procedure used to collect the data is indicated in the standard ISO 17475:

1. sampling of the specimen;
2. measurement of the surfaces and the weight of the sample;
3. immersion for a stated time in the aggressive solution, imposing also the temperature and the concentration of HCl;
4. cleaning and brushing of the specimen to remove all the corrosion products;
5. new measurement of the weight;
6. computation of the change in weight and, consequently, the calculation of the corrosion rate.

The measurement error was conservatively set equal to 5%.

**Figure 1:**
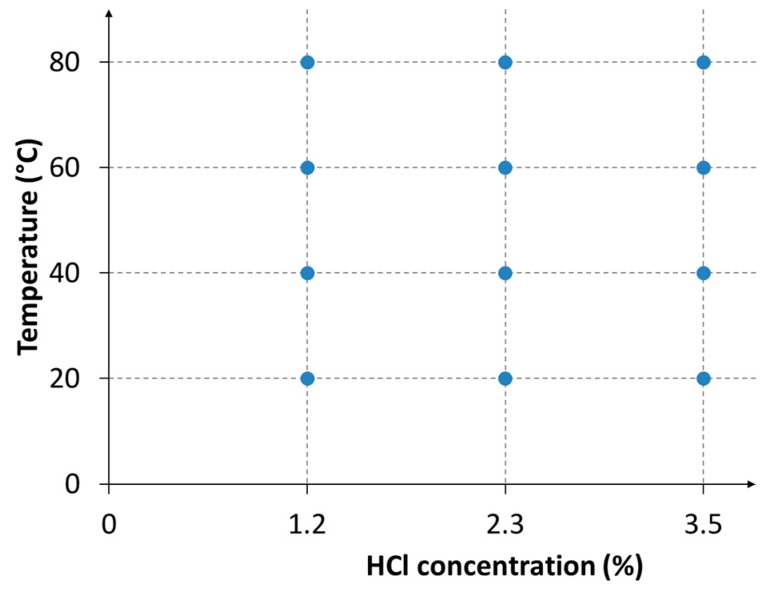
Experimental plan for the study of the corrosion in Chlorine-rich environments, for as-cast and 1300 °C solution-treated samples.

Since the influence of the microstructure plays a key role in the corrosion resistance, especially in Chlorine-rich environments, firstly the influence of the solution treatment temperature was studied, and the behavior of each solution treated samples was studied at a fixed concentration of HCl (2.3%) and temperature (room temperature—RT). Further, the experimental plan (Figure 1) was designed using three HCl concentrations and four temperatures, to derive an iso-corrosion curve that could be directly compared to those of the stainless steels. For each point of the experimental plan, the corrosion rate was determined. This experimental plan was followed for specimens built by the as-cast material and solution treated at 1300 °C, to point out the possible improvement that could be achieved by appropriate heat treatment. Table 1 summarizes the experimental conditions.

To obtain some qualitative information about which is the microstructure that can better withstand the chloride corrosion, solution treated at 1300 °C sample was soaked in an aqueous solution at 3.5% HCl at room temperature for 15 min. Then, their surfaces were slightly polished to remove the surface corrosion products, and these specimens were observed by the optical microscopy.

To give a more complete description of the corrosion resistance behavior of MANC alloy, potentiodynamic polarization tests were performed. The analysis was performed on the as-cast and solution treated at 1300 °C samples. The potentiodynamic curves were obtained using a “three-electrode” potentiostat (AMEL, Milan, Italy) working with a saturated calomel electrode (S.C.E.) in a solution of water and 35 g/L of NaCl. The reference standard for the execution of the tests was the ISO-International Organization for Standardization 17475, using specimens previously polished and left in contact with the atmosphere for 1 day to allow the complete development of any passive film on their surface.

**Figure 2:**
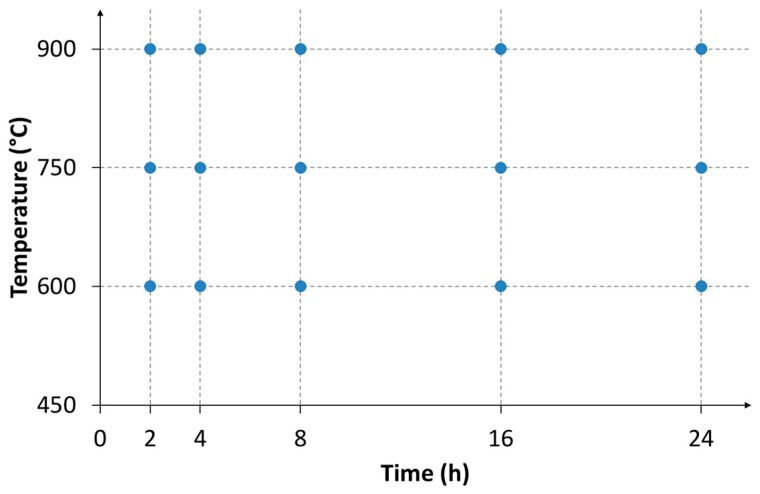
Experimental plan for the study of the hot oxidation resistance for the as-cast alloy.

A different experimental plan was carried out to analyze the two determining factors in the case of the hot oxidation resistance: the exposure time and the temperature. In particular, three temperature levels and five exposure times were investigated, as reported in Figure 2. Air was used at atmospheric pressure as the oxidizing atmosphere. The reference standard applied for the tests was the ISO 21608, which provided the method to evaluate the weight gain on the specimens having the same area. The contact between the sample and support during their permanence in the oven was minimized. All the specimens were shaped in parallelepiped form, starting from the as-cast material; the sizes were measured by a caliber. The final cooling was performed in the air for all the samples. First, the effects of the two variables on influence (exposure time and temperature) were evaluated separately, then, using the obtained regression equation, they were taken into account simultaneously.

To observe which microstructure points out the highest oxidation resistance and if preferential areas of growth of the oxide are present on the surface (i.e., the interface between the phases), a different analysis was performed. Specimens, already undergone to the previously described test, were prepared for the optical microscopy analysis; then, they oxidized again at the same temperature for a soaking time of ten min.

## 3. Results

### 3.1. Metallographic Characterization before Corrosion Test

**Figure 3:**
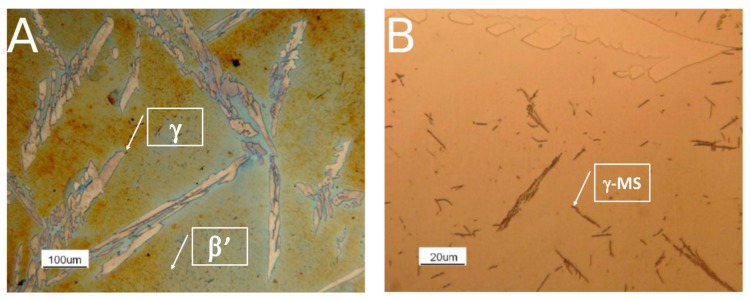
Optical Microscopy microstructure of the as-cast alloy. (**A**) beraha’s tint etching; (**B**) nital 5% etching.

First of all, the metallographic characterization of as-cast and solution-treated samples, before the corrosion and hot oxidation tests, was reported. As-cast alloy was duplex steel formed by β’ matrix and needle-shaped austenite grains (Figure 3A). Within the β’ matrix, small acicular grains of γ-MS (austenite formed by martensite shear phenomena) could be visible at high magnification (Figure 3B). As the Ni concentration is higher compared to typical Fe-Al-Ni steel [6], β’ phase amount was preponderant with respect of γ. About γ-MS, these small acicular grains were non-preferentially oriented and, at high magnification, assumed the typical rhomboidal shape of the non-diffusive growth phase.

**Figure 4:**
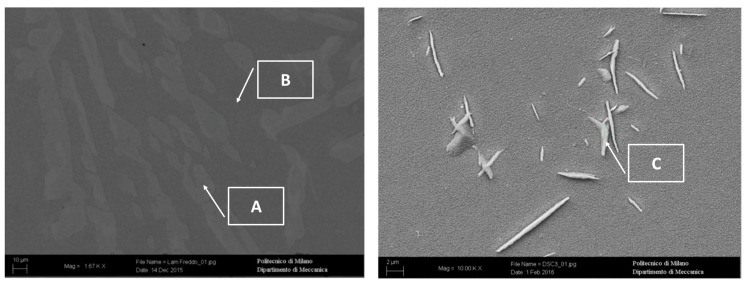
Scanning electron microscopy (SEM) analysis of the as-cast alloy.

SEM images, coupled with EDS spectra, confirmed the nature of the above-mentioned phases (Figure 4, Table 2). As reported in [6], Mn was confirmed to be a γ-stabilizer alloying, whereas Al and Ni promoted the formation of β’. Fe and Cr were mostly in γ-phase and not in the β’ matrix, confirming that the matrix was not ferritic. Martensite shear precipitates had a chemical composition in agreement with γ-phase, confirming that the precipitates were nucleated within the β’ phase grains.

**Figure 5:**
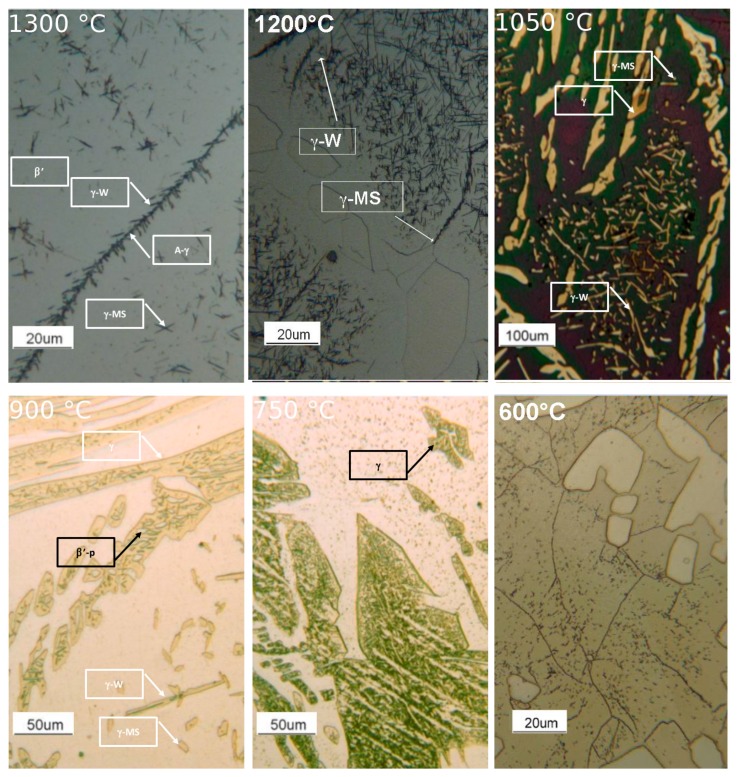
OM of solution-treated samples.

To understand the evolution of the phases, by varying the solution treatment temperature, the microstructure obtained after the solution annealing are reported in Figure 5.

**Figure 6:**
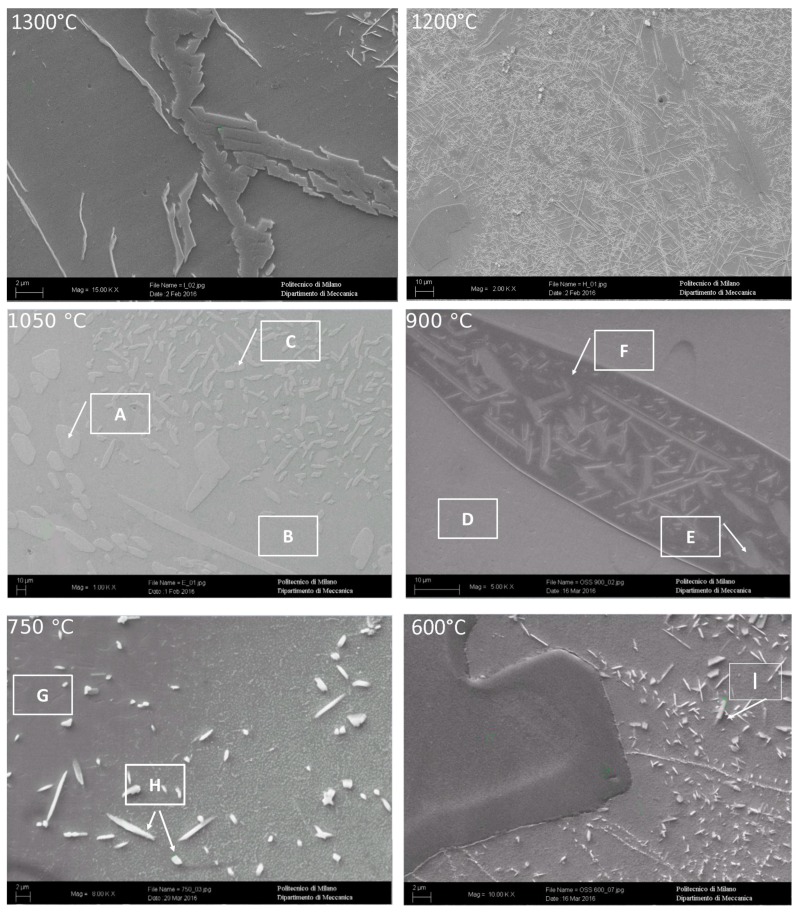
SEM analysis of solution-treated samples.

At 1300 °C, the microstructure was formed by a continuous β’-matrix with allotriomorphic austenite (A-γ) at its grain boundaries, from which type B primary Widmanstätten austenite (γ-W) originated. Fine acicular precipitates (5–10 μm) within the β’ matrix could be also observed. In particular, the acicular precipitates were of martensite shear austenite (γ-MS). These precipitates nucleated from the non-metallic inclusions within the steel. A precipitation free zone, close to β’-matrix sub-grains could be detected. In this sample, the fraction of austenite (A-γ + γ-W + γ-MS) was at its minimum equal to 1% (Figure 5 and Figure 6). At 1200 °C, the microstructure was formed by a continuous β’-matrix with coarse austenite (A-γ) at its grain boundaries. Many acicular precipitates, ordered and located at 90° of each other, were embedded in the matrix, and some precipitation free zone (PFZ) were detected around the residual austenite. After solution treatment at 1050 °C, the MANC alloy remained substantially biphasic, with a higher amount of γ-phase (28%) that appeared in three different morphologies. Typical coarse needle-shaped grains of γ were placed at the β’ matrix grain boundaries. Other γ precipitates, with two different morphologies, were placed within the β’ matrix but far enough from its grain boundaries to define a precipitation free zone (PFZ). Some of the γ precipitates have a pronounced elongated morphology and possess preferential orientations (i.e., Kurdjumov-Sachs [19]). This γ phase could be classified as type D Widmanstätten austenite (γ-W) and originated from the β’ grain boundaries. The remaining γ precipitates could be defined as a martensite shear austenite (γ-MS) since their shape was more regular and did not show any preferential orientations. At 900 °C (after 24 h of solution treatment), austenite still had the three morphology already observed for 1050 °C solution-treated sample: coarse needle-shaped γ grains, γ-MS, and γ-W precipitates. Differently, β’-phase precipitated (β’-p) in the coarse γ-grains. At 750 °C (after 24 h of solution treatment), the morphology of the MANC alloy appeared quite different from that at higher temperatures. Micro- and sub-micrometric precipitates could be highlighted in the β’ matrix, as well as oriented precipitates could still be detected in the coarse γ-grains. The presence of γ-MS and γ-W could be excluded. Due to their small size, the nature of such precipitates could not be precisely identified by OM analysis, but high magnification SEM analysis was required. PFZ was not more visible, hence confirming that the precipitation within the matrix was of a different origin than before. At 600 °C, some precipitates were detected in the matrix; the shape suggested them to be σ-phase. This statement would be confirmed by SEM. 

By SEM/EDS analysis (Figure 6, Table 3), the different phases described above were confirmed. In particular, at high magnification, in sample solution annealed at 1300 °C, allotriomorphic austenite (A-γ) was detected. At 750 °C, the EDS analysis allowed the micro- and sub-micrometric precipitates within the β’ matrix (H point in Figure 6) to be recognized as σ phase, and Al_3_Ni_5_ precipitated. On the other hand, in the sample annealed at 600 °C, σ-phase precipitated into the β’ matrix (I point in Figure 6) due to the high presence of Cr and Mn in the subgrains formed during the treatment.

### 3.2. Corrosion Test 

**Figure 7:**
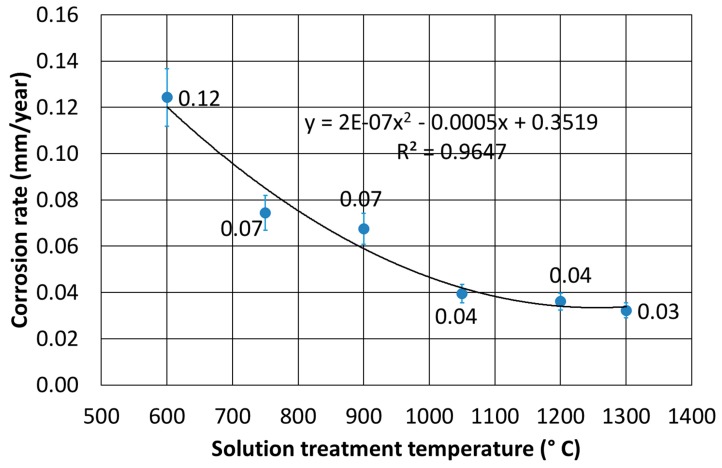
Corrosion rates in 2.3% HCl solution at RT as a function of the solution treatment temperature.

The results of the corrosion resistance at RT, as a function of the solution treatment temperature, are displayed in Figure 7. A strong decrease in the corrosion rate was observed as the solubilization thermal treatment temperature increased, particularly evident in the range 600–1050 °C. The test conducted, in the same conditions, showed the corrosion rate of the as-cast material of about 0.1 mm per year. This indicated that the best condition to put in service this material was in the solution-treated condition.

The iso-corrosion curves were derived, to have a clear understanding of the corrosion phenomenon in the Chlorine-rich environment as a function of both temperature and HCl concentration.

**Figure 8:**
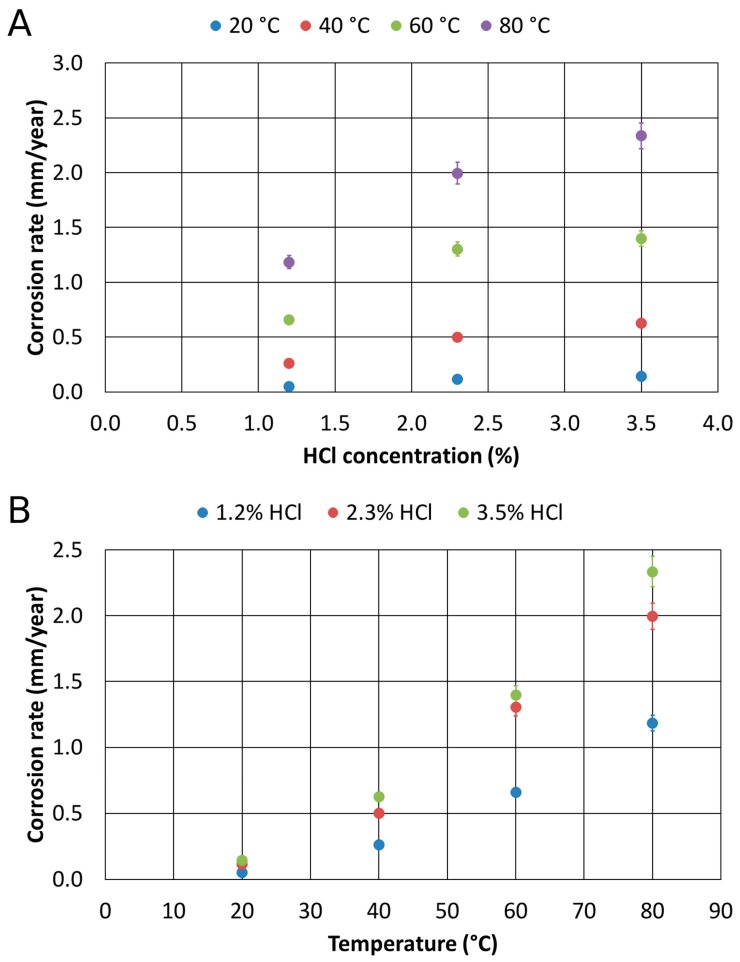
Corrosion rates of the as-cast material as a function of HCl concentration (**A**) and temperature (**B**).

The results obtained for the as-cast material (Figure 8) showed the trends of the corrosion rate as a function of the concentration of HCl at a constant temperature, and then as a function of temperature at a constant HCl concentration: the higher the temperature and the Chlorine concentration, the higher the corrosion rate. 

All the variations of the corrosion rate as a function of the HCl concentration tended to follow a quadratic inverse trend. Thus, it was possible to observe that the effect of HCl concentration gradually became less important, tending to a saturation value. On the other hand, the trends of the rate of corrosion were rather linear as a function of the temperature. Consequently, a strong influence of the temperature on the corrosion rates was highlighted, especially as HCl concentration increased.

Based on the initial part of the curves, it was possible to observe the growth of the initial angular coefficients. Indeed, among the curves in 1.2% and 2.3% HCl, a similar increase of the initial angular coefficient was observed, but its increase in the curves between 2.3% and 3.5% HCl was very small. 

To be able to observe in a single graph the combined effects of temperature and concentration of HCl, the regression of the entire experimental plan was computed and $R^{2}$ (coefficient of determination) value of the regression resembled 99%:

$$\begin{array}{ll} \mathrm{As}-\mathrm{cast}\;\mathrm{material}\; & \mathrm{Corrosion}\;\mathrm{Rate}\\ & =-0.0123-\;0.2251\;\mathrm{HCl}+0.0576{\;\mathrm{HCl}}^{2}+0.01074\;\mathrm{HCl}\;T\;\\ & +\;0.000088{\;\mathrm{HCl}\;T}^{2}-0.002569{\;\mathrm{HCl}}^{2}\;T \end{array}$$

where As−cast material Corrosion Rate corresponds to the corrosion rate of the as-cast material, expressed in mm per year; T is the temperature in °C; HCl is the HCl concentration weight percentage.

**Figure 9:**
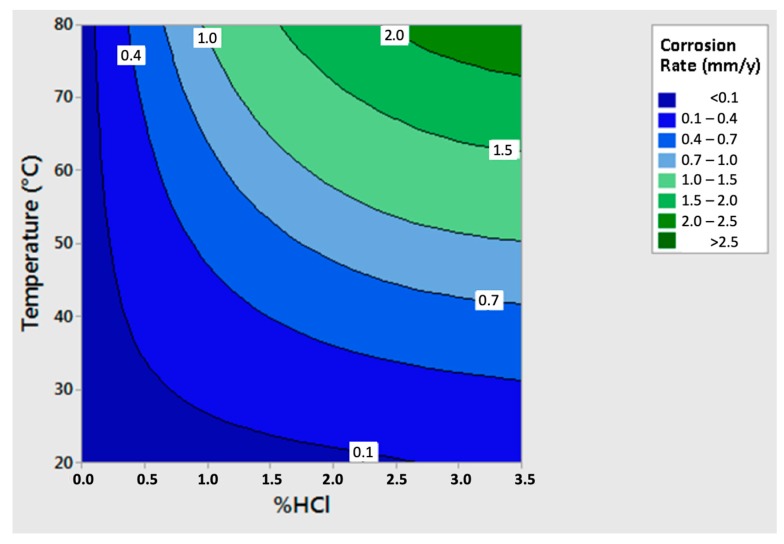
Corrosion rates of the as-cast material as a function of temperature and HCl concentration.

The Equation was also represented in the three axes diagram (Figure 9), where several iso-corrosion lines were highlighted.

**Figure 10:**
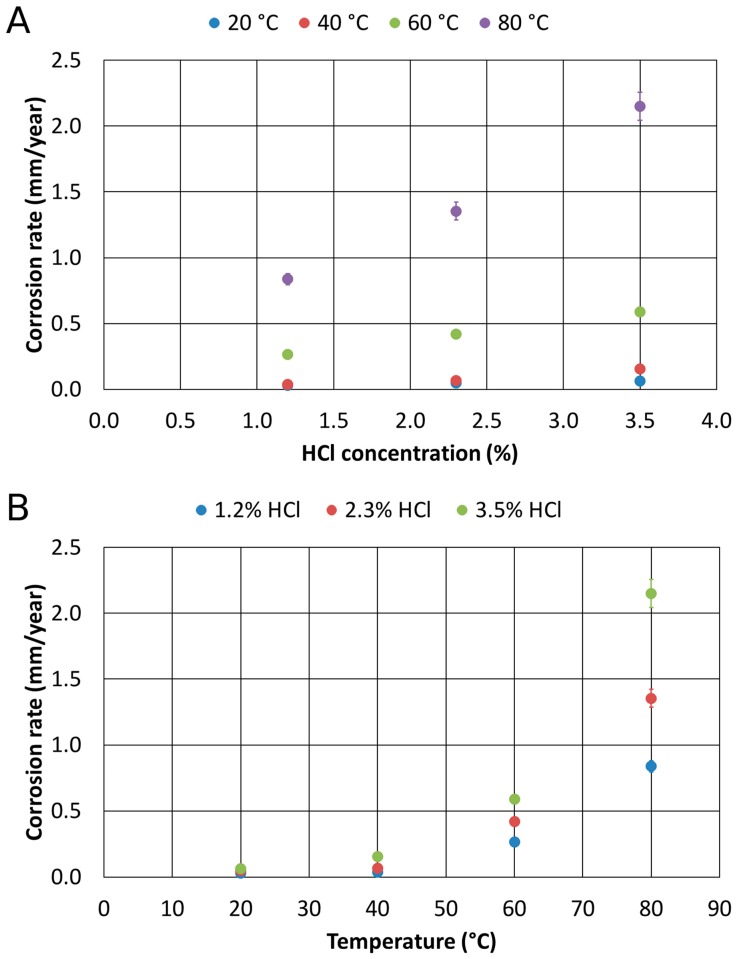
Corrosion rates of the 1300 °C solution-treated alloy as a function of HCl concentration (**A**) and temperature (**B**).

The corrosion resistance tests in Chlorine-rich environments of the 1300 °C solution-treated alloy provided different results. Also, for the solution-treated material at 1300 °C, the charts show, respectively, the corrosion rate as a function of the concentration of HCl at a constant temperature, and then as a function of temperature at constant HCl concentrations (Figure 10).

Based on these results, the strong decrease in the corrosion rate appeared evident, whenever the temperature exceeded 60 °C. All the measured values were quite low, and they indicated that this thermally treated alloy was featured by high corrosion resistance properties. Further, all the corrosion rate trends identified as a function of the HCl concentration and temperatures pointed out a linear behavior. However, the tests performed at 80 °C showed a much lower corrosion resistance if compared to the other collected data. It is worthy of mention that the corrosion rate, at 80 °C and 3.5% HCl, of the solution-treated sample was about the same for the as-cast sample. This aspect could be related to the fact that over 60 °C, the environment is too aggressive, independently from the microstructural feature. This implied also the identification of a critical threshold value, the so-called critical pitting temperature that rules the active-passive corrosion behavior of the 1300 °C solution-treated alloy. This feature appeared more pronounced in the thermally-treated alloy than in the as-cast material. 

As for the previous case, to be able to observe the combined effects of temperature and HCl concentration, the regression of the entire experimental plan was computed, and a regression featured by $R^{2}$ value equal to 98% was stated:

$1300\;{}^{\circ}C\;\mathrm{annealed}\;\mathrm{Corrosion}\;\mathrm{Rate}=0.0193+0.1643\;\mathrm{HCl}-0.01212\;\mathrm{HCl}\;T+\;0.000222{\;\mathrm{HCl}\;T}^{2}$ where 1300 °C annealed Corrosion Rate corresponds to the corrosion rate of the 1300 °C solution-treated alloy, expressed in mm per year; T is the temperature in °C; HCl is the HCl concentration weight percentage.

**Figure 11:**
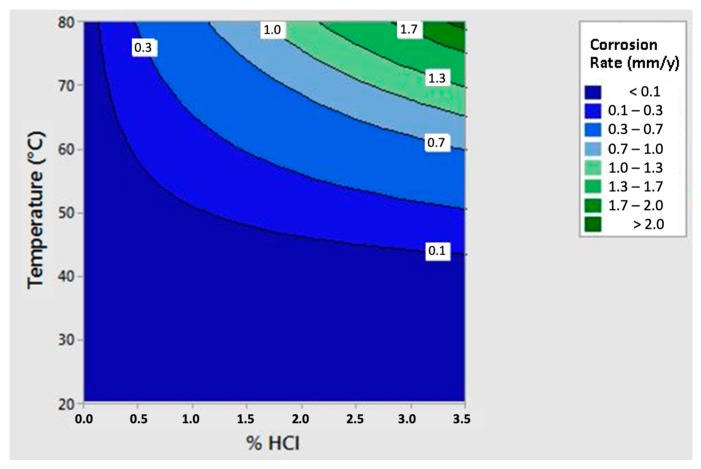
Corrosion rates of the 1300 °C solution-treated alloy as a function of temperature and HCl concentration.

The equation was then represented in the three axes diagram (Figure 11), where several iso-corrosion lines were highlighted.

**Figure 12:**
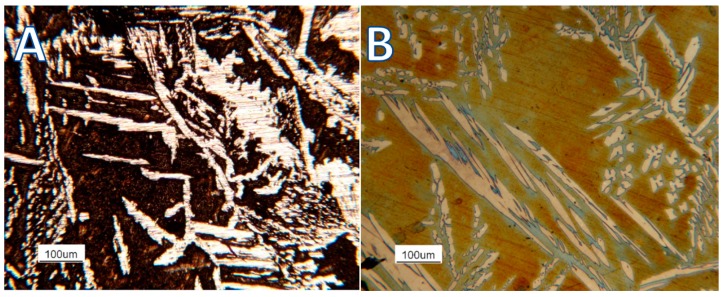
As-cast sample after (**A**) and before (**B**) corrosion test in 3.5% HCl solution at RT for 15 min.

The experimental trials allowed to determine the best structural constituent in terms of the corrosion resistance (Figure 12).

The steels were featured by a duplex microstructure featured by the simultaneous presence of austenite and ferrite. Indeed, the austenitic phase displayed a higher corrosion resistance with respect to the b.c.c. (body cubic centred)β’ matrix. The micrographs showed the b.c.c. β’ matrix deeply corroded by the testing environment. On the other hand, the unaltered austenitic phase morphology showed its much higher corrosion resistance.

**Figure 13:**
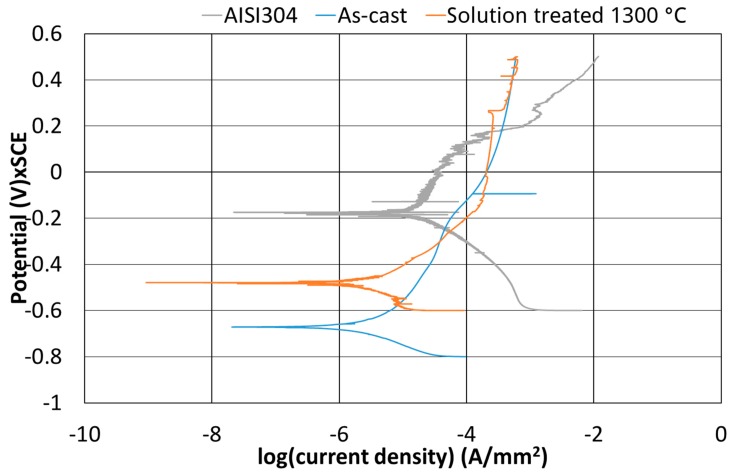
Anodic polarization curves (in 3.5 wt.% of NaCl) of AISI304 (as reference), Fe-Mn-Al-C in the as-cast condition and solution treated (1300 °C for 1 h/inch).

Moreover, the potentiodynamic polarization tests were performed, and the results are reported in Figure 13 and Table 4. These results of the tests allowed to do a comparison among the AISI 304 austenitic stainless steel, the as-cast low-density alloy, and the low-density alloy solution treated at 1300 °C. The initial potential value was considered as the difference between the potential value of the electrode and the reference electrode potential that is featured by a potential of 0.244 V.

### 3.3. Hot Oxidation Tests

**Figure 14:**
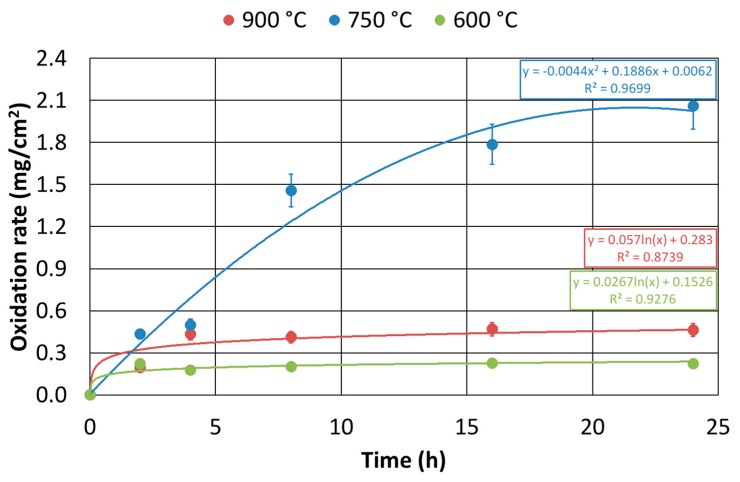
Isothermal curves of the hot oxidation rates over time for the as-cast material.

About the hot oxidation resistance characterization of the as-cast alloy, the experimental plan previously presented was performed, and it aimed at analyzing the time interval of 0–24 h and the temperature range between 600 °C and 900 °C temperature. The results, in terms of isothermal oxidation rate, were measured (Figure 14).

The tests performed at a temperature of 600 °C showed a good agreement with what would be expected from a theoretical point of view. Because of the high concentration of the alloying elements resistant to the hot oxidation, i.e., Nickel, and the relatively low temperature, a logarithmic trend of weight gain as a function of time was pointed out. At 750 °C, the weight increase due to hot oxidation was significant, and it was featured by a sharp decrease in the resistance to this phenomenon. The trend, in this case, turned out to be parabolic, showing a difference of an order of magnitude of the oxidation rate compared to the tests conducted at 600 °C. At 900 °C, the results showed oxidation rates slightly higher than those observed for the tests at 600 °C. There was an order of magnitude difference in oxidation rate between the tests at 600 °C and 900 °C and those performed at 750 °C. 

To highlight the combined effect of exposure time and temperature, a regression equation was obtained and was featured by a $R^{2}$ value of 90%:

$$\Delta m=0.201-1.984\;t+0.005484\;\mathrm{Tt}-0.000004{\;T}^{2}t$$

Δm corresponds to the hot oxidation rate of the as-cast alloy, expressed in $\mathrm{mg}/{\mathrm{cm}}^{2}$; T is the oxidizing temperature in °C; t is the exposure time, expressed in h.

**Figure 15:**
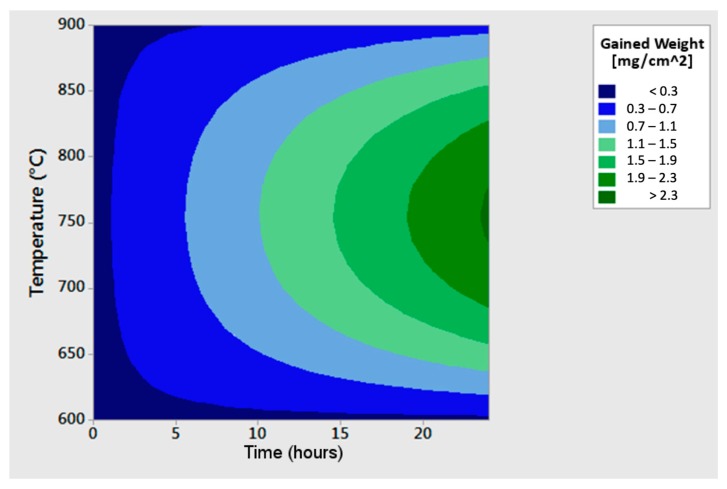
Three axes diagram showing the trend of hot oxidation rates of the as-cast alloy as a function of time and temperature.

This Equation was then represented in a three axes chart (Figure 15).

At 750 °C, a significant increase in the hot oxidation rate occurred even for short exposure times. On the other hand, the behavior, at 600 °C and 900 °C, was very similar. The oxidation rate did not increase proportionally with the temperature.

**Figure 16:**
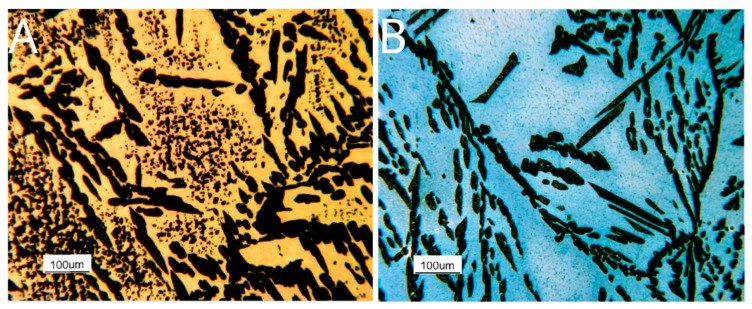
As-cast specimens polished, etched, and hot oxidized for ten min at 750 °C (**A**) and 900 °C (**B**).

To investigate this phenomenon qualitatively, the optical microscopy analysis was fulfilled on the samples chemically etched and oxidized for ten min at the same temperatures at which the tests were carried out, 750 °C and 900 °C (Figure 16). The original microstructure (Figure 16A) and its evolution with temperature have been described in the previous paragraph.

Further, the phases quantification was performed by light optical microscopy, and the obtained data were processed through ImageProPlus software. In the specimen oxidized at 750 °C, 19% of austenite and 16% of σ-phase dispersed within the b.c.c. β’ matrix was present. On the other hand, in the sample oxidized at 900 °C, only the austenitic phase (28%) was detected within the b.c.c. β’ matrix.

**Figure 17:**
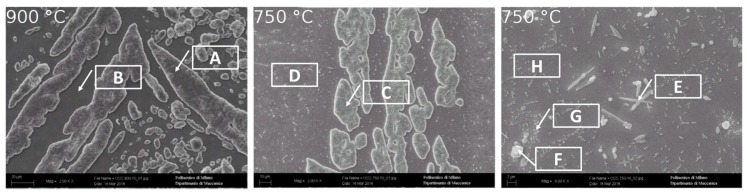
SEM/SE images of the etched and subsequently hot oxidized for 10 min as-cast specimens at 900 °C and 750 °C (γ-phase and β’ matrix detail).

In both specimens, the austenitic phase was oxidized, instead of the b.c.c. β’ matrix, which pointed out different behavior. This result agreed with the chemical analysis, that detected a higher Nickel concentration in the b.c.c. β’ matrix (Figure 17 and Table 5), an element promoting the hot oxidation resistance in the free Sulphur atmosphere. The σ-phase was strongly oxidized in the sample exposed to 750 °C, showing, therefore, a matrix that is, in general, less resistant than that at 900 °C. 

Moreover, the results of the chemical via SEM/EDS for these two specimens were obtained (Figure 17 and Table 5). In both the samples, the austenitic phase appeared as more oxidized than the b.c.c. β’ matrix. The analysis reported a strong presence of Fe and Mn oxides coating the surface of b.c.c. β’ matrix (Figure 17 and Table 5, measures spots A and C). In the sample oxidized at 900 °C, the matrix appeared to be much less oxidized than the austenitic phase. Furthermore, the chemical analysis revealed that the Aluminum oxidized more than the other alloying elements (Figure 17 and Table 5, measures spots B, D, H). Also, in the specimen oxidized at 750 °C, the matrix was not deeply oxidized. However, the σ-phase precipitates were highly oxidized (measure spots E and F). The b.c.c. β’ matrix resulted not to be oxidized in the proximity of the σ-phase precipitates (measure spot G) because of their preferential oxidation and the segregation of Nickel, which acted as a protective element. Other areas of the b.c.c. β’ matrix (which were not protected by the previously mentioned coupled interaction) were oxidized (Figure 17 and Table 5).

## 4. Discussion

The results obtained for the corrosion resistance in Chlorine-rich environments were consistent with the experiments performed on the Fe-Al-Mn-C steel grade [20]. The ferritic phase (and therefore the b.c.c. β’ phase at a higher concentration of Aluminum) always underwent preferential corrosion in the case of a corrosive environment featured by the presence of chlorides. This occurred regardless of the weight concentration of the other alloying elements present in the steel. The reason for this behavior was related to the poor resistance to Chlorine-rich environments of Aluminum, and even the addition of Chromium as a stabilizer b.c.c. β’ phase did not point out any favorable and significant effect to increase the corrosion resistance against the chlorides. Then, the more a phase was rich in Aluminum, thus, the less it resisted to corrosion promoted by chlorides. Consequently, the austenitic phase in the analyzed steel grade would display a higher corrosion resistance in Chlorine-rich environments than the β’phase.

On the other hand, the trend in the corrosion resistance displayed by the solution-treated specimens was in contrast to the previous statement. Indeed, the specimen thermally treated at 1300 °C showed a higher corrosion resistance because the microstructure was composed of the highest volume fraction of b.c.c. β’ phase. This behavior could be explained by the higher chemical and microstructural homogeneities of the 1300 °C solution-treated alloy if compared to the other specimens. The chemical elements and the microstructure at such high treatment temperature underwent a strong solution effect, avoiding and even recovering segregation and precipitation phenomena occurring at lower thermal treatment temperatures. This homogenization effect played a key role in the corrosion resistance behavior of this alloy, ensuring the continuity and the stability of the passive layer at the metal surface.

**Figure 18:**
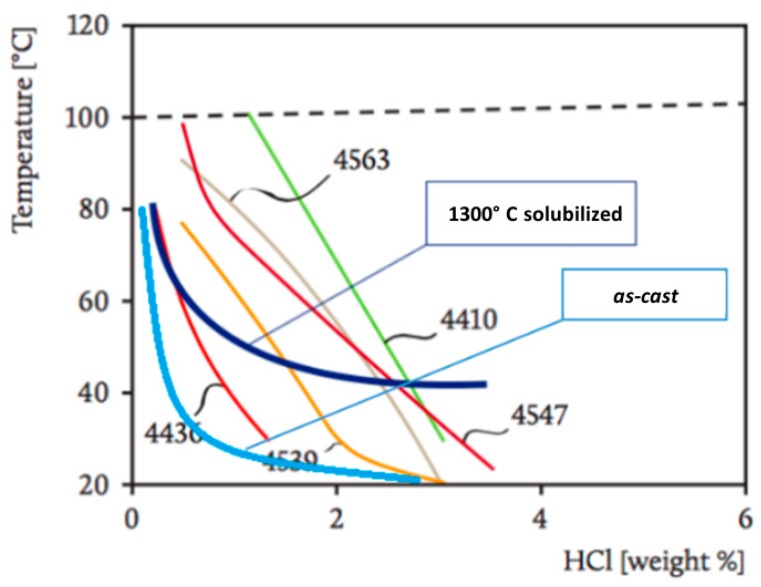
Iso-corrosion curves for the as-cast material and the thermal treated at 1300 °C alloy, compared with several commercial stainless steel grades; it is possible to appreciate the beneficial effect of the high-temperature solution treatment on the analyzed material.

Besides, the iso-corrosion curves of the *as-cast* material and the solution treated at 1300 °C alloys could be compared to those of several commercial stainless steel grades (Figure 18).

The corrosion resistance in Chlorine-rich environments of the as-cast material appeared to be worse than that of the commercial stainless steels. However, although the corrosion resistance at high temperatures (60–80 °C) was quite low, at low temperatures (40 °C) (in particular at room temperature), the corrosion resistance was excellent and competitive against those of the stainless steels, showing values similar to AISI 304. In accordance with this behavior, the slope of the iso-corrosion curve remained low as the concentration of HCl increased, demonstrating the significant influence of the temperature.

The effect of the solution treatment at 1300 °C could be summed up as a considerable increase of the corrosion resistance compared to the as-cast material, in particular at low temperatures (20–40 °C). At high temperatures (60–80 °C), the benefit was still present, but it was less evident, reflecting the great influence of temperature in the corrosion process. At low temperatures (20–40 °C), the resistance was excellent, and it competed with the best stainless steels already available on the market. However, as the temperature increased, the corrosion resistance worsened systematically. The progressive and rapid deterioration of the corrosion resistance was detectable at 60 °C. Therefore, it was probable that this temperature value coincided with the breaking of a passive film and then with the critical pitting temperature. As a consequence, it was possible to divide the iso-corrosion curve of the 1300 °C solution-treated alloy into two areas. In the first, indicatively below 60 °C, the corrosion rate was primarily driven by the temperature, and it displayed a very low slope. On the other hand, above 60 °C, the slope increased drastically, and the HCl concentration resulted as the ruling factor of the corrosion rate. Therefore, this steel ensured exceptional performance in the corrosion resistance, resulting as a potential rival for commercial stainless steels and as a promising material for several industrial applications.

To achieve a more complete description of the corrosion behavior of this alloy, potentiodynamic polarization measurements were performed. The results, reported in Figure 11, showed that the solution treatment increased the corrosion potential of the alloy and after that, the as-cast condition was corrosion activated, and the solution-treated condition showed a passivation zone. The passivation current (Figure 13) of the low-density steel was slightly higher for the solution-treated alloy. In the same way, the corrosion potential was higher for the traditional stainless steel AISI304L, but the transpassivation potential was higher for the investigated alloy in the solution treated condition. 

The best corrosion response of the solution-treated condition was probably due to the microstructure, in particular, the absence of a big amount of secondary phase enhanced the corrosion resistance if compared to the as-cast one. The traditional stainless steel pointed out a better corrosion resistance than the low-density steel, but the difference in the corrosion current was limited (Table 4. Further investigation would be needed for a good comprehension of the mechanisms ruling the corrosion resistance, the features of a protective layer, and the pitting mechanism [21].

The results of the hot oxidation resistance of the *as-cast* material were summarized in the previous chart (Figure 15) and displayed a significant increase of the oxidation rate even for short exposure times at 750 °C. On the contrary, its behavior at 600 °C and 900 °C was very similar, showing a good hot oxidation resistance. The tested material was featured by a particular behavior: the oxidation rate did not increase proportionally to the temperature. The reason for this unusual behavior lied in the absence of the σ-phase precipitates observed in the phase β’ of the sample oxidized at 900 °C if compared to the specimen oxidized at 750 °C. Indeed, it is well-known that the more a phase is pure (therefore, free from precipitates or other phases), the greater is its resistance to hot oxidation [22]. Further, at 600 °C, the low oxidation rate was associated with the low testing temperature.

**Figure 19:**
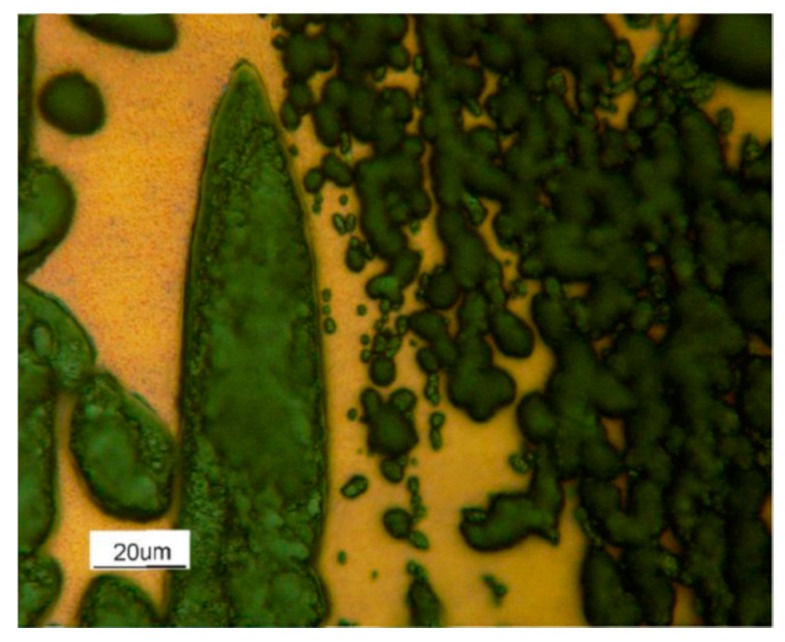
As-cast sample hot oxidized at 900 °C: the detail of the unaltered b.c.c. β’ matrix and the oxide layer born from the austenitic phase.

This behavior could be qualitatively verified through the observation of the optical microscopy results for the samples chemically etched and subsequently oxidized for just ten min. The micrograph of the sample oxidized at 900 °C displayed the detail of the matrix that had not been oxidized and an oxide layer that covered the austenitic phase (Figure 19). The observation of the β’ precipitates, occurring in the austenitic phase at 900 °C, was impossible; the oxidation of the austenite was so severe that it covered and hid the β’ precipitates.

**Figure 20:**
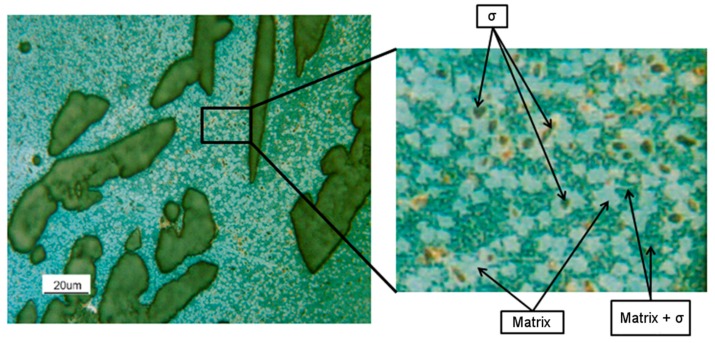
As-cast sample hot oxidized at 750 °C with the high magnified detail of the matrix.

On the other hand, the samples oxidized at 750 °C showed a more complex behavior (Figure 20). σ phase precipitates, here present in a content up to 16%, appeared very oxidized. Within the b.c.c. β’ matrix, two areas could be identified: areas not oxidized in the neighborhood of the precipitates of the σ phase and the degraded areas at larger distances from them. This behavior of the b.c.c. β’ matrix was consistent with a Nickel segregation phenomenon, induced by the σ phase precipitation, which enriched the surrounding area of the b.c.c. β’ matrix with Nickel. Finally, also, the austenitic phase was severely oxidized. Probably, the precipitation of a Cr + Mn rich phase impoverished the β’ matrix of passivizing elements, thus reducing its resistance against hot oxidation. Besides, s phase being enriched in highly oxidizable Mn implied a higher oxidation rate at 750 °C compared to 900 °C. 

Such heterogeneity in the material microstructure decreased considerably the overall hot oxidation resistance [22,23].

The SEM/EDS analysis was performed on these samples (Figure 17 and Table 5) to detect the oxidation attitude of the different phases. The results obtained for the sample oxidized at 900 °C indicated that in the austenitic phase, the Iron and Manganese oxidized more easily than other elements, while in the case of the b.c.c. β’ matrix, the element most prone to the oxidation was the Aluminum.

These results were consistent with those obtained by Trindade et al. [24] for a low-density Fe-Al-Mn-C steel grade. Trindade et al. [24] proved that the oxide formed from the austenite phase was mainly composed of manganese oxide, while the oxide generated by the ferritic phase was alumina. Moreover, the progressive breakage of the alumina (that is formed in the austenitic phase) was related to the manganese oxide volumetric expansion during its formation. Thus, in the steel grade analyzed in this work, the small concentrations of Manganese in the ferritic phase prevented the breakage of the protective oxide layer.

The addition of small quantities of Chromium is favorable to increase hot oxidation resistance in Fe-Al-Mn-C steel grades since it promotes the mechanical resistance of the alumina passive layer [25,26]. However, in the steel grade studied in this work at 750 °C, the presence of Chromium contributed to generate a massive presence of σ phase (formed by Chromium and Manganese), which caused the significant deterioration of the hot oxidation resistance. 

The only differences detected in the microstructure of the as-cast material oxidized at 750 °C and 900 °C were the precipitation of the σ-phase in the specimen oxidized at 750 °C and the increase of the austenitic phase reaching the highest observed volume fraction due to the thermodynamic equilibrium at 900 °C. Thus, the differences in the hot oxidation resistance were associated with these microstructural constituents. Provided the different natures of these phase transformation phenomena, a partially military (without diffusion) austenite transformation and a completely diffusive σ-phase nucleation were observed. Moreover, since the increase in austenite volume fraction at 900 °C was faster than the σ-phase precipitation at 750 °C, at the shortest hot oxidation exposure times (up to 2 h), the hot oxidized sample at 900 °C showed a corrosion rate higher than that at 750 °C. However, at the longest exposure times (24 h), the austenitic volume fraction approached the equilibrium value, and the still present σ-phase led to a continuous increase of the oxidation rate at 750 °C compared to what measured at 900 °C.

**Figure 21:**
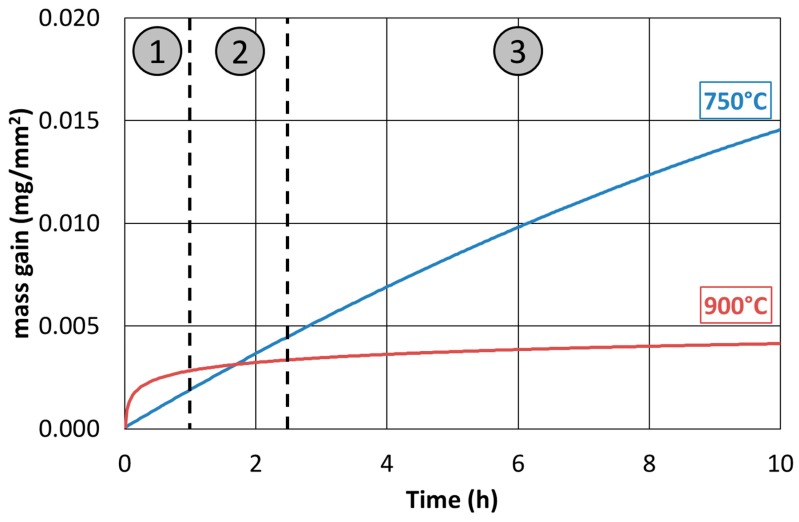
The three oxidation zones of the hot oxidation behavior at 900 °C and 750 °C of the Fe-15%Mn-9.5%Al-6.5%Ni-1%Cr-0.43%C alloy.

The curves of 750 °C and 900 °C oxidation rates evolution could, therefore, be interpreted according to three oxidation steps (Figure 21). In the first step, the oxidation of the austenitic phase and the b.c.c. β’ matrix ruled the total oxidation rate, and the contribution of the σ-phase at 750 °C was small since the σ-phase was yet to be formed in massive quantities. In the second step, severe oxidation began at 750 °C, since σ-phase precipitated and its high Manganese concentration made it very prone to hot oxidation (at about 2 h of exposure time, the total oxidation rates at 750 °C and 900 °C were equal). In the third step, the oxidation rate at 750 °C exceeded the one at 900 °C, due to the fact that the σ-phase that was formed at 750 °C gave a very high contribution to the total oxidation rate, which was greater than that of the newly generated austenite phase at 900 °C.

Finally, the collected results showed the opportunities for the use of the Fe-15%Mn-9.5%Al-6.5%Ni-1%Cr-0.43%C alloy, where the hot oxidation resistance is a crucial aspect. The results at 600 °C and 900 °C showed very good resistance to oxidation, and the values were competitive with those found for the family of austenitic stainless steels grades. However, at 750 °C, a significant difference compared to the performance of stainless steels at the same temperature was recorded; this was related to the decrease of hot oxidation resistance associated to the σ-phase precipitation (Figure 3, Figure 4, Figure 17 and Figure 20). Thus, the results, in terms of performance, were comparable to those of the austenitic stainless steels grades at working temperatures outside the range of the σ-phase precipitation. This result is interesting because some previous studies [27] on the hot oxidation resistance of the Fe-Al-Mn-C steel grades have not shown any temperature range where they could be competitive if compared to the traditional Chromium-based stainless steels. The addition of Nickel was proved to be beneficial for hot oxidation resistance properties.

Therefore, in typical applications of the steels resistant to this phenomenon (such as furnaces and industrial reactors, facilities for combustion processes), a common practice is the use of common steels up to 550 °C, Chromium-Molybdenum steels up to 650 °C, Chrome-Molybdenum-Silicon alloyed steels up to about 750 °C, stainless steels up to 900 °C, and Nickel-based superalloys for the highest working temperatures. Thus, MANC steels could replace the above mentioned high-alloyed steels with benefits in term of lightness and production costs, by adding Al and replacing Ni with Mn.

## 5. Conclusions

In this work, the corrosion resistance and the hot oxidation resistance of the Fe-15%Mn-9.5%Al-6.5%Ni-1%Cr-0.43%C alloy (produced by laboratory tests as low-density steel grade) were characterized via different methods.

The corrosion resistance in Chlorine-rich environments was evaluated both through iso-corrosion curves (derived by gravimetric measurements) and through potentiodynamic polarization analysis. It was verified that the austenitic phase was more resistant than the b.c.c. β’ matrix due to the lower presence of Aluminum, which has poor resistance to Chlorides. The iso-corrosion curves showed good corrosion resistance for the as-cast material, comparable to austenitic stainless steels at low temperatures, and excellent resistance of the 1300 °C solution-treated samples, especially at low temperatures but also good at high temperatures. Also, the potentiodynamic tests showed a clear improvement of the performances after the solubilization thermal treatment performed at 1300 °C, and a corrosion resistance behavior comparable to the AISI 304 austenitic stainless steel was pointed out.

The hot oxidation resistance, analyzed at 600 °C, 750 °C, and 900 °C, showed a rate of oxidation comparable to that of austenitic stainless steels at 600 °C and 900 °C. However, at 750 °C, a clear worsening of the hot oxidation resistance occurred due to the massive precipitation of σ-phase (16%), which has extremely poor resistance against the dry oxidation.

The results obtained for this Fe-15%Mn-9.5%Al-6.5%Ni-1%Cr-0.43%C alloy are promising for the exploitation of this steel grade in those applications where corrosion resistance in Chlorine-rich environments or hot oxidation resistance are relevant. 

## Figures and Tables

**Table 1:** Summary of experimental conditions realized on MANC alloy.

Batch n°	Solution Treatment Temperature (°C)	HCl Concentration (%)	Corrosion Temperature (°C)	Exposure Time (h)
1	600; 750; 900; 1050; 1200; 1300	2.3	RT	24
2	As-cast; 1300	1.2; 2.3; 3.5	20; 40; 60	24
3	As-cast	-	600; 750; 900	2; 4; 8; 16; 24

**Table 2:** Energy dispersive spectroscopy (EDS) analysis of the as-cast alloy reported in Figure 4.

wt.%	Fe	Cr	Ni	Mn	Al
A (γ)	67.03	1.22	5.33	17.22	8.66
B (β’)	63.30	0.87	8.62	14.28	11.88
C (γ-MS)	67.03	1.26	6.67	15.84	9.20

**Table 3:** EDS analysis of solution-treated samples in Figure 6.

Solution Treatment Temperature (°C)	wt.%	Fe	Cr	Ni	Mn	Al
1050	A (γ)	68.58	1.27	4.82	17.63	7.70
	B (β’)	62.23	1.21	6.41	14.54	9.60
	C (γ-MS)	67.80	1.43	4.72	18.45	7.61
900	D (β’)	68.24	0.93	6.53	14.14	10.15
	E (γ)	68.16	1.27	3.40	19.97	7.20
	F (β’-p)	65.91	1.06	7.14	16.09	9.80
750	G (β’)	68.57	0.73	5.93	15.03	9.74
	H (σ)	39.73	17.54	5.46	29.99	7.29
600	I (σ)	50.75	12.93	3.65	27.77	4.90

**Table 4:** Potential and Current values derived from the anodic polarization curves (Figure 9).

-	Corrosion Potential (E_corr_) [V]	Transpassivation Potential (E_pp_) [V]	Passivation Current (I_p_) [A/mm^2^]
AISI304	−0.17	0.18	−4.4
As-Cast	−0.67	-	-
Solution Treated 1300 °C	−0.48	0.27	−3.6

**Table 5:** SEM/EDS analysis of the etched and subsequently hot oxidized for 10 min as-cast samples at 900 °C and 750 °C (Figure 17).

Hot Oxidation Temperature (°C)	wt.%	Al	Cr	Mn	Fe	Ni	O
900	A (γ)	-	-	43.96	33.88	-	22.16
	B (β’)	18.02	-	12.02	57.91	5.03	7.02
750	C (γ)	-	-	27.42	54.84	-	17.74
	D (β’)	11.77	0.74	14.07	67.31	6.11	-
	E (σ)	7.91	987	24.55	40.30	4.26	13.10
	F (σ)	3.68	-	20.07	51.81	1.81	22.63
-	G (β’ near σ)	11.49	1.09	14.48	63.29	5.95	-
-	H (β’)	12.15	0.92	14.36	66.74	5.83	3.70
